# Cardiac Pain and Angina Pectoris

**Published:** 1897-11-20

**Authors:** S. H. Habershon

**Affiliations:** Assistant Physician and Pathologist to the Hospital


### Medical Progress and Hospital Clinics.

[*The Editor will be glad to receive offers of co-operation and contributions from members of the profession. All letters should be addressed to* The Editor, at the Office, 28 & 29, Southampton Street, Strand, London, W.C.]

### CARDIAC PAIN AND ANGINA PECTORIS.

A Clinical Lecture and Demonstration given at the Brompton Hospital for Consumption and Diseases of the Chest, by S. H. Habershon, M.D., F.R.C.P., Assistant Physician and Pathologist to the Hospital.

(*Continued from page* 117.)

### The Inducing Causes of an Attack.

The conditions mentioned so far are the more theoretical and ultimate causes of angina. Practically, given an over-irritable vasomotor system, a feeble or imperfectly-nourished heart, or a condition of increased peripheral resistance in the arterioles, we find the inducing causes in those circumstances which (1) increase the tension of blood in the arteries, (2) cause distension or sudden dilatation of the cavities of the heart, and (3) produce a sudden call upon the powers of an enfeebled cardiac muscle. Mental excitement, exertion in any form, stooping, lifting, walking (especially uphill or upstairs), the ingestion of food, and the acceleration of heart and circulation thus produced—these are some of the inducing causes of an attack of cardiac pain.

### Classification.

The difficulty of classification is considerable, because many of the cases overlap, and we find more than one of the conditions I have mentioned present. For practical purposes I have placed them in the following groups, which I shall endeavour as far as possible to illustrate by cases.

I. Cases Arising from Central Causes.

1. Neurovascular spasm.
2. Neurovascular spasm, associated with asthma (possibly pneumogastric in origin).

II. Cases of Peripheral Origin.

1. Purely peripheral. Senile changes in arterioles, causing loss of elasticity and tendency to a high tension pulse on slight provocation. Sometimes (especially when associated with a feeble cardiac muscle) producing angina pectoris sine dolore, more rarely cardiac pain. Sometimes fatal.
2. Peripheral, due to condition of blood, gouty cases, in early stages, leading later to atheroma or changes in the cardiac muscle, and entering into the next group.

(*The above central and peripheral cases are comprised in the angina pectoris vasomotoria of Nothnagel.*)

III. Cases of Cardiac Origin.

1. Idiopathic an.ina pectoris, due to disease of coronary arteries, or to degeneration of cardiac muscle. May be brought about by any cause, peripheral or otherwise, which leads to degenerative changes in heart, aorta, or coronary vessels.
2. Cardiac valvular disease, aortic stenosis or incompetence, aortic atheroma. Usually leading to disease and obstruction of coronary orifices, or to imperfect nutrition of a hypertrophied heart.
3. Distension of cardiac cavities in mitral disease.
4. Aneurisms or other tumours pressing on the basal cardiac plexuses (spurious angina or pseudo-angina).

### A.—Angina Pectoris Vasomotoria.

**Vasomotor Angina of Central Origin.**—Just as in certain cases of idiopathic asthma a condition of hypersensitiveness of the bronchioles may be induced, so the whole neurovascular system may be thrown into a condition of spasmodic contraction by influences arising from the central nervous system. This hyperæsthetic state of the vasomotor system is sometimes seen in women of the neuropathic type, though it is not confined to one sex. A vasomotor spasm of the minute arterioles appears to be induced, producing a small, frequent, high-tension pulse and cardiac pain of varying severity. In the earlier stages, there is no cardiac lesion to be found, and the cause of angina is to be sought in the increased tension in the cardiac cavities. These cases were first described by Nothnagel, and his views have been endorsed by Powell, Broadbent, Brunton, and others. The unstable condition of the vasomotor system is exhibited by the slight cause that induces an attack. A chill, or exertion, or excitement, or any factor which produces increase of arterial tension, is sufficient. According to Fagge, Nothnagel describes the onset of an attack as accompanied by coldness, pallor, and numbness of the extremities. The cardiac pain is not always increased by exertion, and in the following patients, whom I show you this afternoon, moderate exercise seems to do no harm.

A. B., aged 30, who comes of a very neurotic family and admits that he is very nervous, came to me as an out-patient a few weeks ago. He is here for your inspection this afternoon. He tells me that for three years past he has suffered from a pain in the chest, not frequent until the last six months. The pain is very severe, and begins in the cardiac area, spreading upwards to the left shoulder and down the inner side of the left arm and thence to the fingers. It is accompanied by coldness of both upper extremities, and always leaves a numb sensation behind. When the attacks first commenced they were preceded by shivering and general chilliness. The attacks are produced by excitement or exertion, and the pain, he tells us, "frightens him."

The pulse is firm, though scarcely at the present time of markedly high tension. When you listen to his heart you will find an accentuated second aortic sound. The size of the heart is normal in every respect, and there are no abnormal sounds except the second aortic. He has no albuminuria.

This is an almost typical case of vasomotor angina pectoris, and corresponds very closely to those described by Nothnagel. You will observe that it is not of long duration, and the heart appears to be vigorous and healthy, the spasm commencing as a purely peripheral and vasomotor storm.

The next case I shall have to show is somewhat similar in origin, but the patient is a woman, now 59 years of age, and the attacks have continued since the age of 18 or 19. It is interesting to notice that in her case the heart has become weak and degenerate, and this emphasises an important point to which Sir R.

Douglas Powell calls attention. It is important, because it leads to some confusion between cases commencing in what may be called a central neurovascular disturbance and the classical type of idiopathic angina pectoris, which is so often of manifestly cardiac origin, and accompanied by disease of the coronary arteries in some cases, and in others by degeneration of the muscular substance of the heart. The fact I refer to is that a continuance of high tension for perhaps many years eventually leads to dilatation or organic disease of the tissues of the heart. In the second case, to which I have just referred, there are signs that this result has occurred. The woman is stout, and it is difficult to define the heart's apex; but you will find that the sounds are heard unusually far out, and though there is no bruit, there is, in addition to some increase of tension of the radial pulse, that peculiar sharpness both of heart-beat and pulse that we are accustomed to associate in stout women over middle age with a weak and flabby left ventricle. This is the explanation I have to offer for the cardiac pain in this case, and for the great increase of intensity during the past few months.

I have a third case, in which I believe the unusual cardio-vascular disturbance to be dependent on a central cause. It is in a highly hysterical young woman. She suffers from spasmodic cardiac pain, but, in addition, there is a peculiar bronchial neurosis. The patient is comfortable in the erect position, but not only the recumbent position, but even the inclination of the head backwards brings on the most violent expiratory dyspnœa. I have never seen a neurosis precisely similar. On placing the patient on the sofa, as I did inadvertently on my first examination, the dyspnœa was most alarming. The pulse became frequent and sometimes irregular, and the heart tumultuous, so that the patient appeared almost in a moribund condition.

(*To be continued.*)

